# Antileukemic activity of novel adenosine derivatives

**DOI:** 10.1038/s41598-019-50509-1

**Published:** 2019-10-01

**Authors:** Anastazja Poczta, Aneta Rogalska, Małgorzata Łukawska, Agnieszka Marczak

**Affiliations:** 1 Department of Medical Biophysics, Institute of Biophysics, Faculty of Biology and Environmental Protection, University of Lodz, Pomorska 141/143, 90-236 Lodz, Poland; 2 Synthesis and Chemical Technology Department, Łukasiewicz Research Network-Institute of Biotechnology and Antibiotics, Staroscinska St., 02-516 Warsaw, Poland

**Keywords:** Haematological cancer, Toxicology

## Abstract

The present study investigated the effect of cladribine (CLA) and six of its derivatives containing a formamidine group at position 6 (CLA-FDM, CLA-FPAZ, CLA-FPIR, CLA-FPIP, CLA-FHEX, and CLA-FMOR) on acute promyelocytic, lymphoblastic, and acute monocytic leukemia cells. The role of ATR kinase in deoxycytidine kinase (dCK) activation in response to DNA damage was assessed. The presence of DNA lesions was assessed by measurement phosphorylation of H2AX and by using the alkaline comet assay with proteinase K post-treatment following assessment of the cell cycle. Apoptotic events such as alterations in intracellular calcium concentration, caspase-3/7 activity and increased sub-G1 cell population were measured. CLA derivatives were highly effective against leukemic cells, showing high cytotoxicity, causing DNA fragmentation, and inducing DNA-protein cross-links in leukemic cells. CLA-FMOR showed the highest efficacy. CLA derivatives increased the levels of intracellular calcium ions, caspase-3/7 and the percentage of sub-G1 apoptotic cells and blocked cells in the S phase of the cell cycle to a greater extent than free CLA. The selective ATR inhibitor VE-821 significantly suppressed the increase in dCK activity and decreased basal dCK activity. The present results suggested that ATR kinase controls dCK activity in response to synthetic CLA derivatives.

## Introduction

Hematopoietic malignancies are currently one of the most common causes of death among patients of various age groups. A chemotherapeutic agent that is currently used to treat lymphoid malignant tumors is cladribine (CLA), which is approved by the Federal Drug Administration for hairy cell leukemia, Langerhans cell histiocytosis (LCH), and other lymphoproliferative disorders^1^. Purine nucleoside analogs, such as cladribine or fludarabine in combination with monoclonal antibodies (Rituximab or Alemtuzumab) and cyclophosphamide are currently the gold standard for the treatment of leukemia^2^. Cladribine is not only used in oncology, but also for the treatment of other disorders based on its efficacy in the treatment of multiple sclerosis^3^. Cladribine, as well as 2′-deoxyadenosine is transported inside the lymphocyte through human equilibrative nucleoside transporter. CLA is resistant to deamination by adenosine deaminase (ADA) due to the presence of a chlorine atom in the structure of the purine ring, but is efficiently phosphorylated by dCK and 5′-nucleotidases (5′-NT) to 2-chlorodeoxyadenosine monophosphate (2-CdAMP) and then to triphosphate 2- chlorodeoxyadenosine (2-CdATP). The intracellular concentration of these phosphorylated purines is regulated by deoxycytidine kinases and 5′-nucleotidases. Selective 2CdA toxicity to lymphocytes is due to high dCK activity and low 5′-nucleotidase activity^3,4^. These compounds function through a similar cytotoxic mechanism in both proliferating and resting cells^5^. Cladribine, with the participation of p53 protein and proteins from the Bcl-2 family, induces the apoptotic intrinsic pathway of apoptosis^6,7^. Nucleoside analogs inhibit ribonucleotide reductase and reduce the amount of deoxynucleotides in the cell. Cladribine modifies level of histones, inhibits the action of anti-apoptotic proteins, reduces the mitochondrial membrane potential, increases the production of reactive oxygen species in cells and causes intracellular calcium growth, resulting in the release of apoptosis-inducing factors such as cytochrome C, second mitochondria-derived activator of caspases/direct IAP-binding *protein* with low PI, and apoptosis-inducing factor^4,7,8^. Cladribine also promotes arrest of the cell cycle in the G_2_/M phase, condensation of chromosomes and DNA fragmentation. Cladribine induces apoptosis by accumulation of double-stranded DNA breaks and by increasing the level of γH2AX^9,10^^,^. The first step of activating cladribine is catalyzed by deoxycytidine kinase (dCK). This enzyme is mainly expressed in lymphocytes, whereas cladribine is particularly active in lymphoid tissues^11^. Genotoxic agents, including UV-C and DNA synthesis inhibitors or cladribine contribute to increase of ATR (Ataxia Telangiectasia and Rad3-related protein) kinase activity, which is a major activator of dCK in leukemic cells^5,12^. ATR participates in response to single-stranded (ssDNA) and double-stranded DNA breaks (DSBs) and a variety of DNA lesions that interfere with replication^13^. ATR promotes cell cycle arrest and repair of DNA or induces apoptosis if the repair systems are overwhelmed (activating CHK-1 kinase and phosphorylating many proteins that are part of the DDR pathway: H2AX, BRCA1/2 (breast cancer type 1/2 susceptibility protein), RAD51 and p53)^14^.

The aim of the present study was to elucidate the mechanism of action of cladribine derivatives using acute monocytic leukemia (THP-1), acute promyelocytic leukemia (HL-60), and acute lymphoblastic leukemia (MOLT-4) cell lines as a model, and to compare their genotoxic and cytotoxic properties to those of the parent drug, cladribine. Six new derivatives of cladribine (CLA-FMOR, CLA-FPIR, CLA-FPIP, CLA-FHEX, CLA-FDMF, and CLA-FPAZ) were analyzed. The role of ATR in dCK activation in response to cladribine derivatives was also investigated.

## Results

### Cytotoxic assay *in vitro*

**Figure 1:**
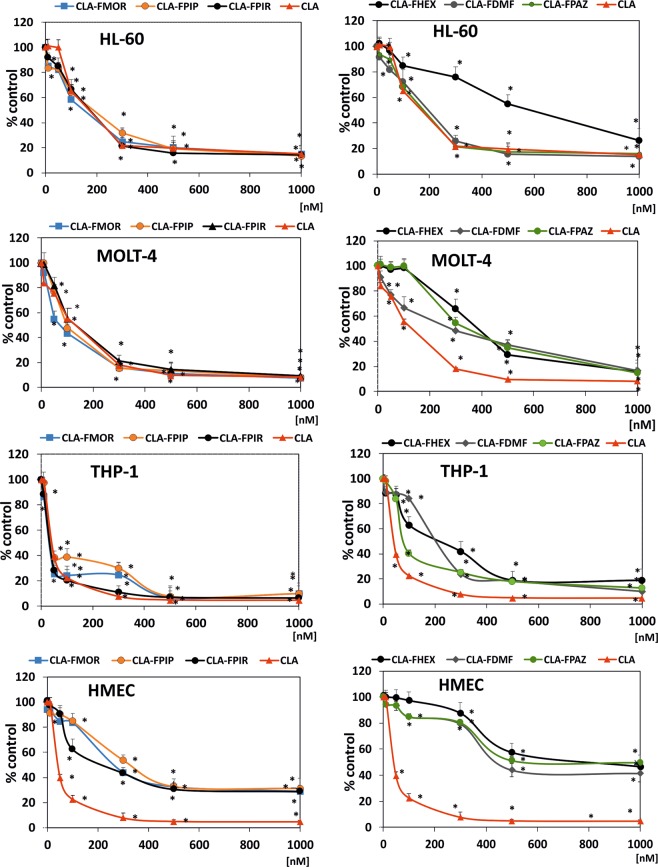
Concentration dependent cytotoxic effect of cladribine (CLA) and its derivatives (panel left: CLA-FMOR, CLA-FPIP and CLA-FPIR; panel right: CLA-FHEX, CLA-FDMF, CLA-FPAZ) on HL-60, MOLT-4, THP-1 and HMEC-1 cells growth measured by XTT assay. Cells without any treatment were used as controls and taken as 100%. Results are presented as the mean of six independent experiments. Error bars signify standard deviation, * indicate statistically significant changes between samples incubated with the drugs compared with control cells (P < 0.05). Statistical analysis was performed by ANOVA test with the Tukey post hoc test for multiple comparisons.

**Figure 2:**
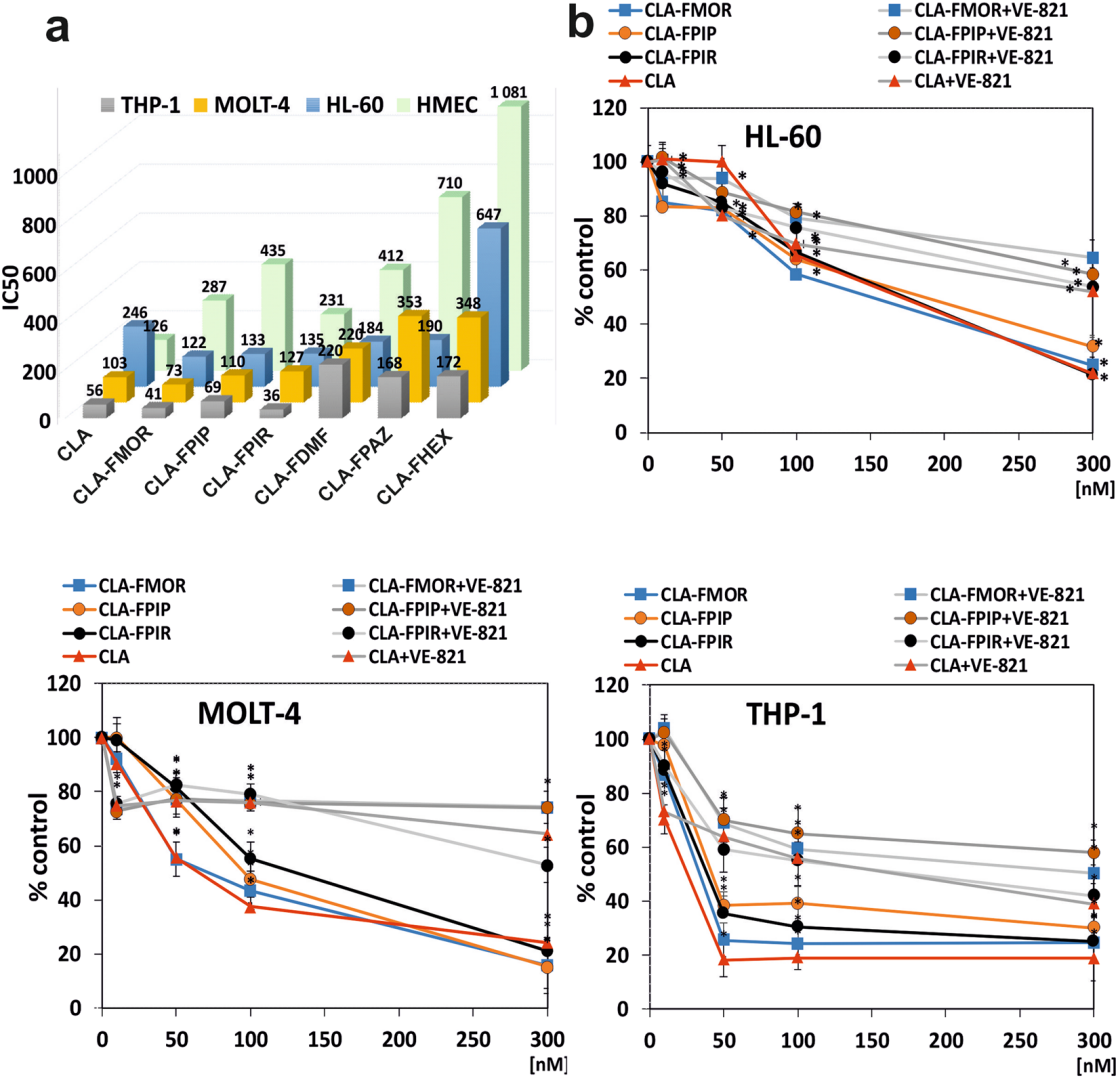
(**a**) Comparison of the cytotoxicity (designated as IC50) of cladribine (CLA) and its derivatives in HL-60, MOLT-4, THP-1 and HMEC-1 cell lines. (**b**) Effect of ATR inhibitor (VE-821) on cytotoxicity of CLA and its derivatives. For comparison the effect of VE-821 cells were preincubated with or without 10 μM VE-821 and cells viability was measured by the XTT assay after 48 h of incubation. Samples without VE-821 are presented as color lines and samples with VE-821 as gray lines. Cells without any treatment were used as controls and taken as 100%. Results are presented as the mean ± SD of six independent experiments. Error bars signify standard deviation, * indicate statistically significant changes between samples incubated with the drugs compared with control cells (P < 0.05). Statistical analysis was performed by ANOVA test with the Tukey post hoc test for multiple comparisons.

The cytotoxic activity of the compounds was assessed using the XTT assay. The results for all compounds are presented in Fig. 1. Both, the derivatives and the reference drug showed concentration-dependent cytotoxic effects. The sensitivity to the tested compounds differed among the different cell lines, as indicated by the IC_50_ values (Fig. 2). In HL-60 cells, all derivatives except CLA-FHEX were more cytotoxic than CLA. In MOLT-4 cells, only CLA-FMOR was more potent than CLA. THP-1 cells showed the highest sensitivity to CLA-FMOR and CLA-FPIR. CLA-FHEX, CLA-FDMF, and CLA-FPAZ showed low cytotoxicity and were therefore excluded from subsequent experiments. Doses of 130 nM for HL-60, 80 nM for MOLT-4, and 40 nM for THP-1 cells were used in all subsequent experiments. The derivatives of cladribine were less cytotoxic than the parent drug against the dermal endothelial cell line HMEC-1. We have observed that the ATR inhibitor VE-821 reduces the cytotoxicity of all tested compounds.

### ATR is involved in controlling dCK activity

**Figure 3:**
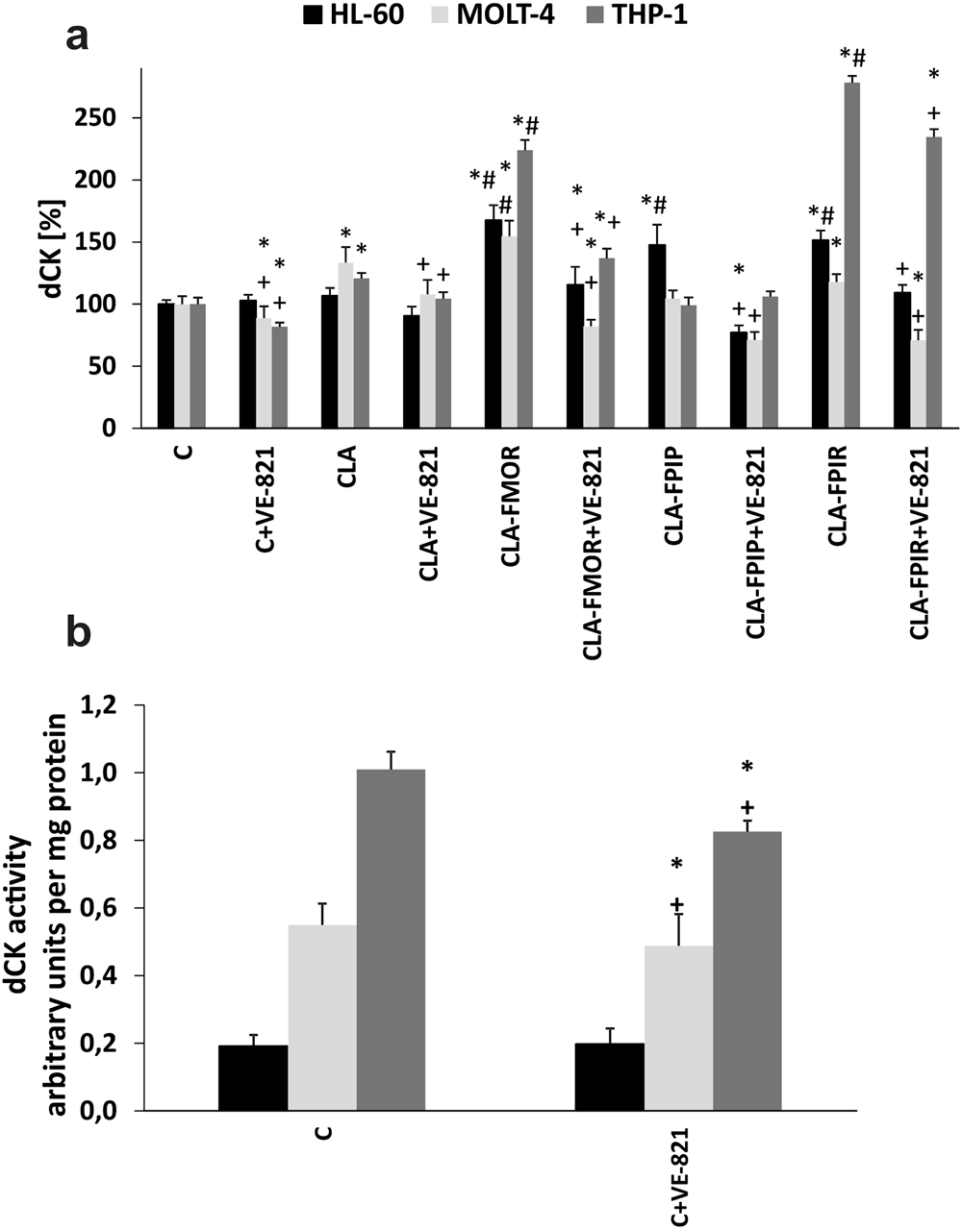
(**a**) The dCK activity in THP-1, MOLT-4, and HL-60 cell lines incubated with cladribine and its derivatives for 24 h in comparison to the activity in control cells. Cells without any treatment were used as controls and taken as 100%. In some experiments (marked on the X axis as: + VE-821), the cells were preincubated with VE-821 for 1 h at 37 °C before addition of the drugs and further incubation for 24 h. (**b**) Differences in the level of basic dCK activity between the tested cell lines. Error bars signify standard deviation, * indicate statistically significant changes between samples incubated with the drugs compared with control cells (P < 0.05). Symbol + indicate statistically significant changes between samples incubated with the compounds and VE-821 or caspase 3 inhibitor (P < 0.05); ^#^ indicate statistically significant changes between samples incubated with cladribine and new derivatives (P < 0.05). Statistical analysis was performed by ANOVA test with the Tukey post hoc test for multiple comparisons.

Cladribine was selectively toxic against leukemic cells because of the high activity of dCK in these cells. The dCK was activated by ATR kinase, which was activated in response to a broad spectrum of genotoxic compounds. The role of ATR kinase in regulating dCK activity was investigated using an ATR kinase (VE-821) inhibitor. The results are shown in Fig. 3. dCK activity was measured after 24 h of incubation with cladribine and its derivatives. We present differences in dCK level between cell lines (Fig. 3b). The highest dCK activity was observed in human acute monocytic leukemia (THP-1) cells after incubation with the CLA-FMOR and CLA-FPIR derivatives. In the HL-60 and MOLT-4 cell lines, the highest dCK activity was observed after incubation with the CLA-FMOR derivative.

VE-821 decreased dCK activity in all cell lines, both under control conditions and after DNA damage induced by CLA, CLA-FMOR, CLA-FPIP, and CLA-FPIR. This suggested that other kinases affected dCK activity. Overall, the results strongly suggested that ATR was involved in the regulation of dCK activity.

### Intracellular distribution of calcium

**Figure 4:**
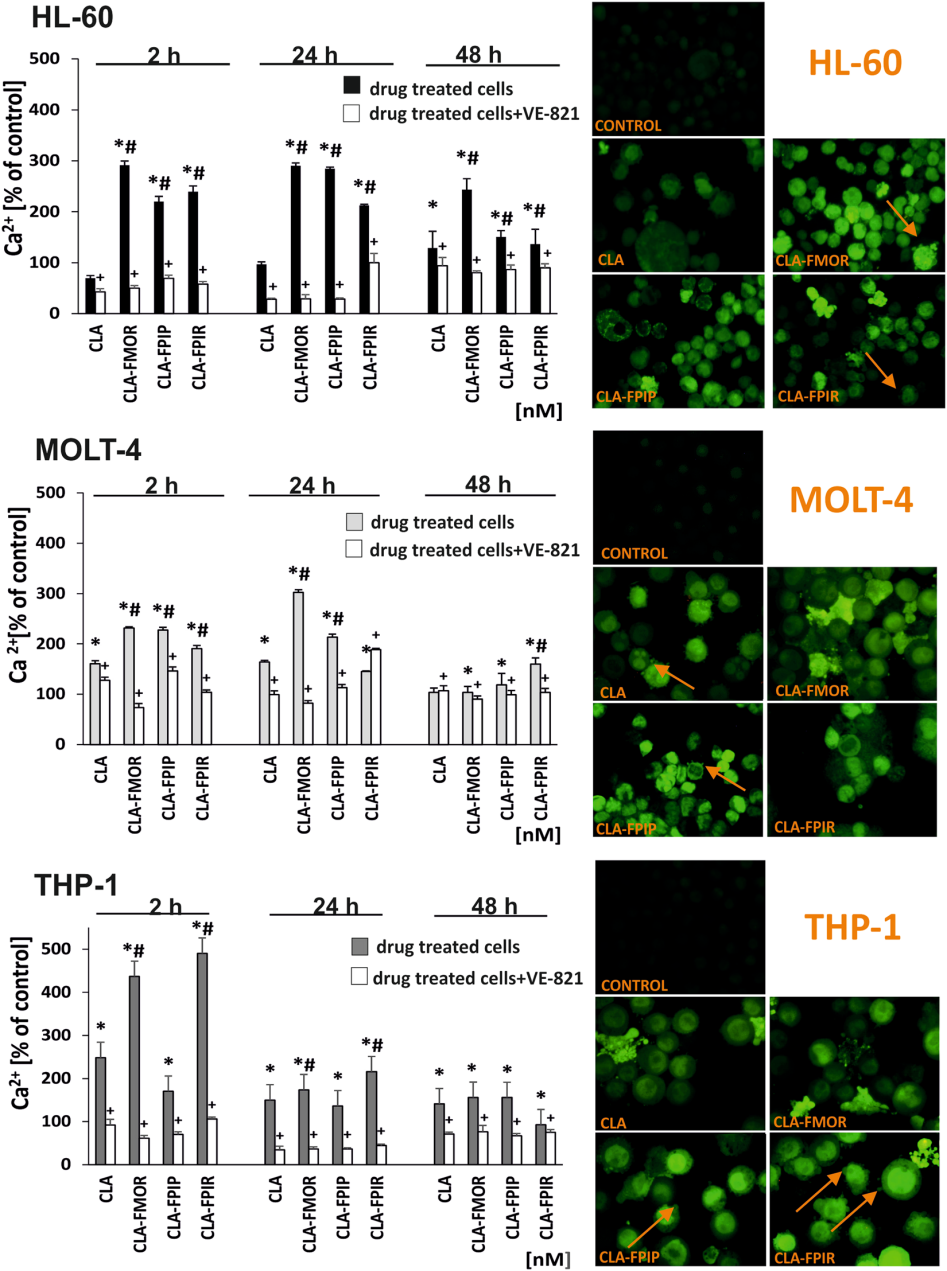
Effect of cladribine and its derivatives on intracellular Ca2 + levels in three cell lines: THP-1, MOLT-4, and HL-60. Cells were incubated with the different compounds for 2, 24, and 48 h. In some experiments (presented as white bars), the cells were preincubated with ATR inhibitor (VE-821) for 1 h at 37 °C before the addition of drugs and incubation for an additional 2–48 h. Cells, after 24 h incubation with compounds, stained with the Fluo-4-AM probe were visualized under an inverted fluorescence microscope (Olympus IX70, Japan; magnification: 400 × ). Some cells display abnormal morphology: cells with protrusions from the plasma membrane (blebbing), marked by the orange arrows. Error bars signify standard deviation, * indicate statistically significant changes between samples incubated with the drugs compared with control cells (P < 0.05). Symbol + indicate statistically significant changes between samples incubated with the compounds and VE-821 or caspase 3 inhibitor (P < 0.05); ^#^ indicate statistically significant changes between samples incubated with cladribine and new derivatives (P < 0.05). Statistical analysis was performed by ANOVA test with the Tukey post hoc test for multiple comparisons.

Ca^2+^ ions play a crucial role in the induction of apoptosis in lymphocytes^15–17^. Fluo-4-AM acetoxymethyl ester was used to determine the intracellular levels of calcium ions. The results presented in Fig. 4. Show fluorescence images of leukemia cells morphology after 24 h incubation with new cladribine derivatives. Cells treated with the tested compounds were visualized and imaged with a fluorescence microscope. The strong green fluorescence in the cells indicated an increase in intracellular calcium levels. Some of the cells have protrusions from the plasma membrane (blebbing) marked by the orange arrows. Cladribine derivatives significantly increased intracellular calcium ion levels in all cell lines compared with the effect of CLA. The highest intracellular calcium ion concentration was observed in THP-1 cells incubated for 2 h with the CLA-FMOR and CLA-FPIR derivatives. HL-60 and MOLT-4 cells showed early changes in the amount of calcium ions after incubation with cladribine derivatives. Treatment with the ATR kinase inhibitor reduced intracellular calcium ion levels in HL-60, MOLT-4, and THP-1 cells after incubation with cladribine and its derivatives independently from incubation time.

### New CLA derivatives increases pro-apoptotic gene expression

**Figure 5:**
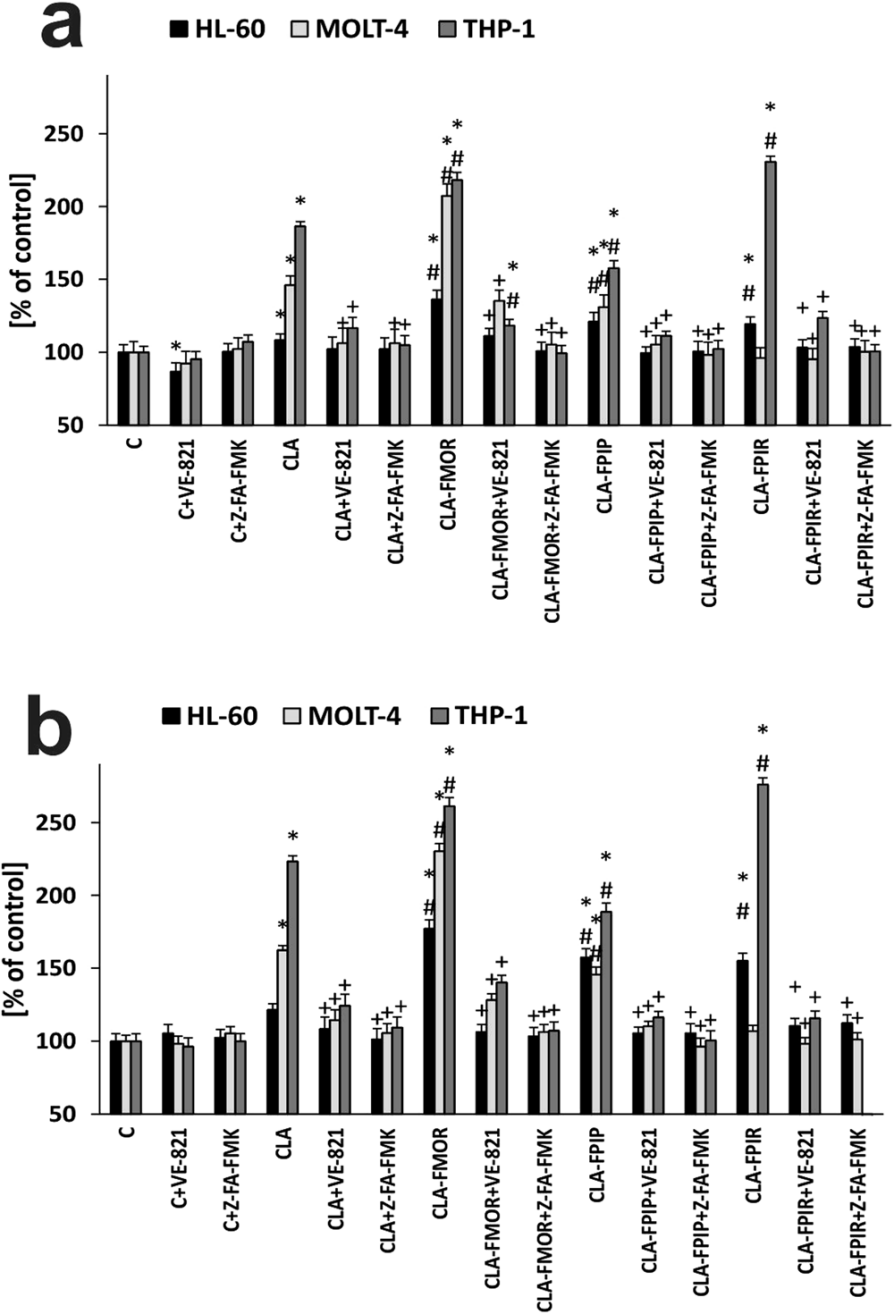
Caspase-3/7 gene expression as an indicator of apoptosis in three cell lines: THP-1, MOLT-4, and HL-60. Cells without any treatment were used as controls and taken as 100%. The cells were treated (**a**) 24 h and (**b**) 48 h with the compounds in the presence or absence of VE-821 and Z-FA-FMK inhibitors. Error bars signify standard deviation, * indicate statistically significant changes between samples incubated with the drugs compared with control cells (P < 0.05). Symbol + indicate statistically significant changes between samples incubated with the compounds and VE-821 or caspase 3 inhibitor (P < 0.05); ^#^ indicate statistically significant changes between samples incubated with cladribine and new derivatives (P < 0.05). Statistical analysis was performed by ANOVA test with the Tukey post hoc test for multiple comparisons.

Caspase-3/7 was analyzed as an indicator of apoptosis, in the presence or absence of ATR inhibitor. The results are presented in Fig. 5. Changes in the level of caspase-3/7 were time-dependent. In all cells the most significant changes in caspase activation were observed after 48 h of compounds treatment. The highest level of caspase was in the THP-1 cell line, which was also the most sensitive cell line to the compounds. After 48 hours incubation with the tested compounds, the highest changes in the HL-60 cell line were caused by the CLA-FPIP and CLA-FPIR derivative, in the MOLT-4 by CLA-FMOR, and in the THP-1 cells by CLA-FPIR. New derivatives lead to higher, statistically significant differences, in the caspase 3/7 level compared to cladribine. According to protective effect of VE-821 observed in cytotoxicity assays, we found that activation of caspase-3/7 in response to cladribine and new derivatives was significantly reduced in the presence of the ATR inhibitor, indicating a decrease in apoptosis. Pre-treatment with the caspase inhibitor, Z-FA-FMK, totally prevented caspase activation, suggesting the involvement of caspase-dependent mechanisms in new CLA derivatives induced cytotoxicity.

### The level of ɣH2AX

**Figure 6:**
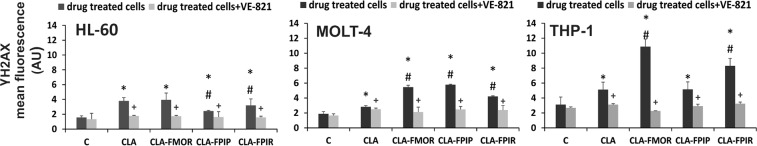
The level of phosphorylation of H2AX (γH2AX) induced by cladribine and its derivatives (CLA-FMOR, CLA-FPIP and CLA-FPIR) in HL-60, MOLT-4 and THP-1 cell lines. Cells were incubated with the compounds for 48 h. In some experiments, the cells were preincubated with ATR inhibitor (VE-821) for 1 h at 37 °C before the addition of CLA or CLA derivatives. Error bars signify standard deviation, * indicate statistically significant changes between samples incubated with the drugs compared with control cells (P < 0.05). Symbol + indicate statistically significant changes between samples incubated with the compounds and VE-821 or caspase 3 inhibitor (P < 0.05); ^#^ indicate statistically significant changes between samples incubated with cladribine and new derivatives (P < 0.05). Statistical analysis was performed by ANOVA test with the Tukey post hoc test for multiple comparisons.

To assess whether CLA derivatives causes DNA damage, the level of phosphorylated H2AX was evaluated. Induction of γH2AX in response to new purine analogs in leukemia was measured after 48 h of compounds incubation with the cells. The ɣH2AX level was markedly higher in all tested probes than in the control cells. The data are additionally indirectly confirmed by the comet assay (detection of DNA-protein cross-links by proteinase K) and by the activation of the executive caspase. We observed a significant ~2.4-fold increase in ɣH2AX level in cells incubated with CLA and CLA-FMOR in HL-60 cells. There was a significant difference between the effect induced by CLA and its derivatives. In THP-1 and MOLT-4 cells new compounds were more or at similar level potent as CLA. In MOLT-4 cell lines CLA-FMOR and CLA-FPIR were about 2-fold more effective in inducing dsDNA than CLA. Also, in the THP-1 cell line there was a 2-fold increase in the level of dsDNA breaks against CLA (Fig. 6). We showed here that increase in the level of γH2AX, induced by cladribine and by drug’s derivatives, were significantly reduced by the ATR inhibitor VE-821.

### Cell cycle distribution

**Figure 7:**
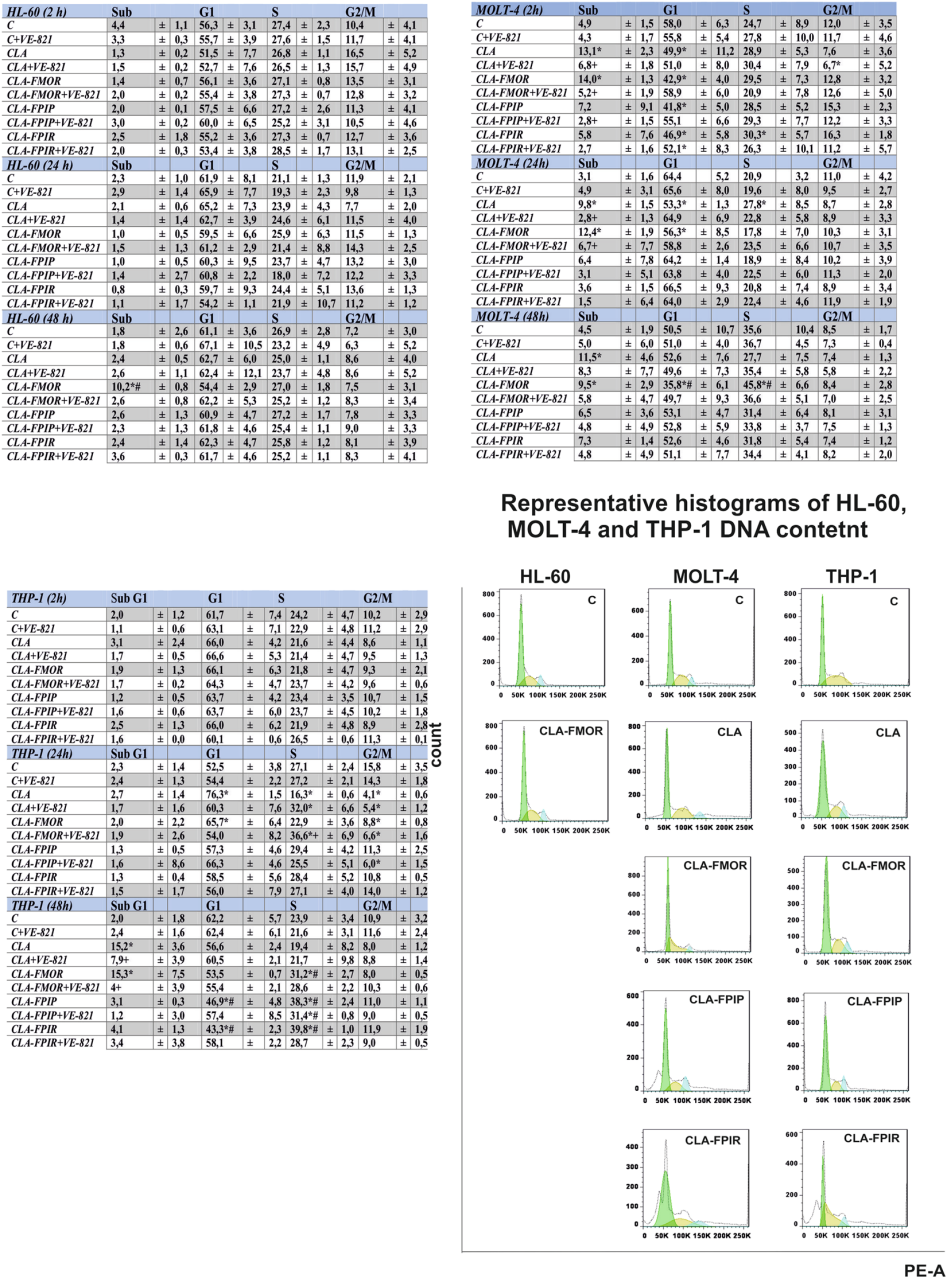
Some cells display abnormal morphology: cells with p Cell cycle phase distribution presented as a percentage share of phase subG1, S, G1, G2/M in HL-60, MOLT-4, and THP-1 cells (2–48 h) treated with the compounds in the presence or absence of the ATR inhibitor (VE-821). Individual phases were identified based on cellular DNA content quantified by flow cytometry. Representative histograms of DNA content after treatment with each compound for 48 h. * indicate statistically significant changes between samples incubated with the drugs compared with control cells (P < 0.05). Symbol + indicate statistically significant changes between samples incubated with the compounds and VE-821 or caspase 3 inhibitor (P < 0.05); ^#^ indicate statistically significant changes between samples incubated with cladribine and new derivatives (P < 0.05). Statistical analysis was performed by ANOVA test with the Tukey post hoc test for multiple comparisons.

The effect of CLA and its derivatives on cell cycle distribution in leukemic cells was analyzed by flow cytometry. The effect on the cell cycle was dependent on the sensitivity of each cell line to the test compounds. CLA and its derivatives had a mild effect on cell cycle phase distribution in HL-60 cells. CLA-FMOR for 48 h was the only derivative that increased the proportion of cells in the sub-G1 population by approximately 10% (Fig. 7).

In MOLT-4 cells, CLA and CLA-FMOR caused an early increase (after 2 h incubation) in the number of cells in the sub-G1 phase. This effect was sustained independently of incubation time. All compounds reduced the number of cells in the G1 phase after 2 and 48 h compared with that in control cells. After 48 h of incubation, there were statistically significant differences between the effects of CLA and CLA-FMOR. CLA-FMOR reduced the number of cells in G1 phase to approximately 36% and blocked the cell cycle at the S phase (46%).

In THP-1 cells, no changes in cell cycle distribution were observed after 2 h of incubation with the tested compounds. No changes in the number of cells in the sub-G1 phase were detected after 24 h of incubation with the new derivatives. The parent compound reduced the number of cells in the S phase and blocked the cell cycle at the G1 phase. CLA-FPIP, CLA-FPIR, and CLA-FMOR stopped the cell cycle at the S phase at 48 h in THP-1 cells, and CLA-FPIP and CLA-FPIR decreased the number of cells in G1 phase (42.37%). VE-821 restored the effects of the drugs in all tested cell lines to the level of control.

### Drug-mediated DNA damage

**Figure 8:**
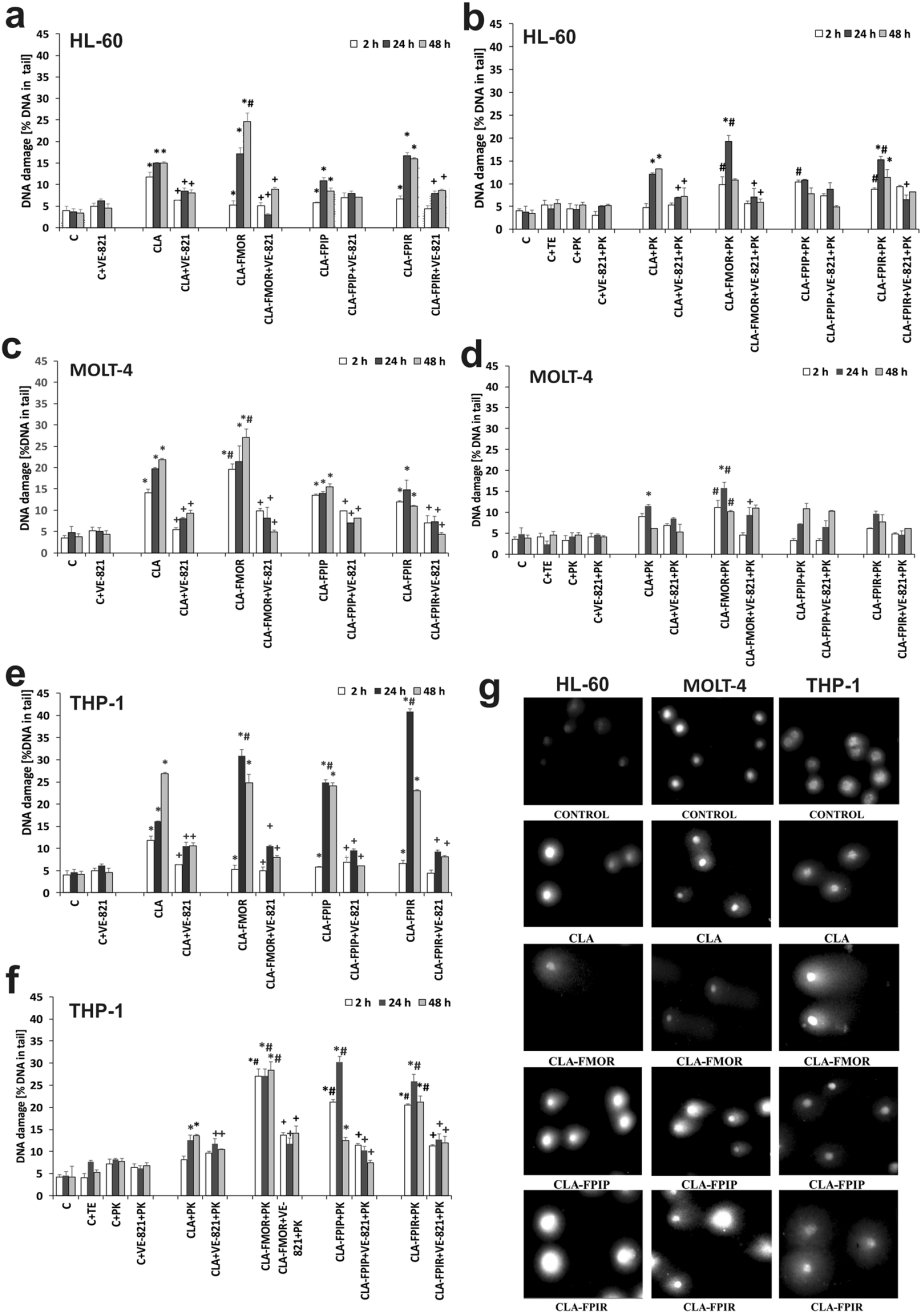
Fragmentation of DNA after treatment the cells with CLA and its derivatives, measured as a percentage of the DNA in the comet tail. Samples were preincubated with ATR inhibitor, VE-821 (**a**,**c**,**e**) or treated with proteinase K (**b**,**d**,**f**) in HL-60, MOLT-4, and THP-1 cells for 2, 24, and 48 h. Representative images of comets. Characteristic comet tails with damaged DNA were visualized under a fluorescence microscope after electrophoresis and gel staining with DAPI. (**g**) Error bars signify standard deviation, * indicate statistically significant changes between samples incubated with the drugs compared with control cells (P < 0.05). Symbol + indicate statistically significant changes between samples incubated with the compounds and VE-821 or caspase 3 inhibitor (P < 0.05); ^#^ indicate statistically significant changes between samples incubated with cladribine and new derivatives (P < 0.05). Statistical analysis was performed by ANOVA test with the Tukey post hoc test for multiple comparisons.

The “alkali labile sites” were analyzed as described above. Figure 8(a,c,e) shows DNA damage measured as a percentage of the DNA in the comet tail of cells incubated for 2, 24, and 48 h. Probes were preincubated with or without VE-821 for 1 h at 37 °C before incubation with the different compounds. The comet assay showed that both cladribine and its derivatives induced DNA damage in leukemia cells in a time-dependent manner. DNA damage in the comet tails increased as early as 2 h after treatment. THP-1 cells showed the highest sensitivity to the tested derivatives. The level of DNA damage was correlated with cytotoxicity. Cladribine induced 16% DNA strand-breaks at 24 h. However, treatment with the derivatives caused a steady increase in DNA damage in comet tails, which reached a maximum value at 24 h for CLA-FMOR (31%) and CLA-FPIR (41%). In the HL-60 and MOLT-4 cell lines, a significant increase in DNA fragmentation (24% and 27%, respectively) was observed after 48 h of incubation with the CLA-FMOR derivative. At 48 h, DNA damage induced by CLA-FMOR in HL-60 cells was two-fold higher than that induced by the unmodified drug. Finally, we investigated whether ATR was also involved in regulating the activity of CLA derivatives in cancer cells. Silencing of ATR significantly decreased genotoxicity regardless of the incubation time.

Figure 8(g) shows representative results of the comet assay in control cells and in cells treated with CLA or its derivatives. Comets originating from the controls had a nearly symmetrical shape and practically no tails, whereas comets from human leukemia cells treated with cladribine analogs showed longer tails. This difference was especially prominent in cells treated with CLA-FMOR.

### Detection of DNA-protein cross-links by proteinase K

Fig. 8(b,d,f) shows the mean length of the comet tails of leukemia cells exposed to CLA and its derivatives after subsequent treatment with proteinase K. The results showed a greater tail length (DNA fragmentation) in cells treated with the enzyme. Incubation of cells with proteinase K increased the level of cross-links in HL-60 cells starting at 2 h of incubation with CLA-FMOR and CLA-FPIP. Extending the incubation time to 24 h with CLA-FMOR and CLA-FPIR in HL-60 cells increased the DNA damage. MOLT-4 cells showed an increase in the percentage of DNA in the comet tail after 24 h of incubation with CLA-FMOR. DNA damage in the tail of THP-1 cells increased after treatment with proteinase K in cells incubated with CLA-FMOR for 2–48 h, CLA-FPIP for 24 h, and CLA-FPIR for 24 h. VE-821 reduced the level of DNA-protein cross-links induced by derivatives at all incubation times.

## Discussion

Purine nucleoside analogues are an important group of highly effective drugs that are used to treat leukemia^18^, as demonstrated in studies of cladribine analogues. The cytotoxicity and pro-apoptotic potential of adenosine modified with the carborane cluster against mononuclear peripheral blood mononuclear cells in patients with leukemia has been investigated^2^. Computer-designed novel derivatives of cladribine, such as 2-amino-2-(4-(6-amino-9H-purin-9-yl)- alkyl) propane-1,3-diol, have been synthesized and characterized^10^.

Cladribine is a prodrug, and its activity depends on the intracellular accumulation of active triphosphate (CdATP). This process depends on the ratio of dCK to 5′-NT and cell type. Data from the BioGPS on mRNA profiling show that dCK levels and dCK to 5′-NT ratios are particularly high in T lymphocytes (CD4+, CD8+), B lymphocytes, and dendritic cells, whereas they are low in many non-hematological cell types, including cells in the liver, heart, skin, brain, lungs, kidneys, ovaries, testes. This results in selective cytotoxicity of cladribine against lymphocytes^19^.

To determine the cytotoxic effect of CLA and its derivatives (CLA-FDMF, CLA-FPAZ, CLA-FPIR, CLA-FPIP, CLA-FHEX, and CLA-FMOR), an XTT reduction test was performed. THP-1, HL-60, and MOLT-4 cells showed statistically significant differences in sensitivity to the tested compounds. The cytotoxic effect of CLA was dose-dependent. Three of the tested derivatives (CLA-FPIR, CLA-FPIP, and CLA-FMOR) showed high cytotoxic activity against leukemic cells. The XTT test does not distinguish between cytotoxic effects of the tested compounds and inhibition of cell proliferation or cytostatic effect. However, it indicates the level of toxicity of new derivatives. These compounds were more cytotoxic than the parent compound and were therefore selected for subsequent experiments. Differences in the structure of the tested compounds concern both the size and the type of the amine ring. The studied derivatives have the rest of 5-, 6- and 7-membered cyclic secondary amines. Some of the tested analogs possess also additional heteroatom- oxygen. So, in explanation of differential cytotoxic activity of the compounds, we should consider both the size of the ring and its geometry. Additionally, we expect that due to the presence of a heterocyclic ring introduced simultaneously into the molecule of cladribine, it is possible to improve the bioavailability and chemical stability of the analogue. This will be the subject of further research for selected analogues. There are reports in the literature about both positive and negative effects of C-6 modification, which is probably crucial for its activity^20,21^. Moreover, other studies indicate that purine nucleoside antimetabolites and their monophosphate derivatives were exported from the cell by the ABC transporter ABCG2. Clofarabine and cladribine are dCK substrate. It was confirmed that ABCG2 inhibitors would effectively increase the anti-tumor efficacy of purine nucleosides by blocking drug efflux which may be a significant of resistance when dCK levels are low^22^. The THP-1 cell line with the highest dCK level is the most sensitive to the new derivatives of cladribine. Further tests are necessary to confirm. The most active compounds in THP-1 cells were CLA-FMOR (IC_50_: 41 nM) and CLA-FPIR (IC_50_: 36 nM). The highest decrease in viability was observed in MOLT-4 and HL-60 cells after incubation with the CLA-FMOR derivative. To determine whether the tested derivatives had selective activity against leukemic cells, the cytotoxicity of these compounds was tested in a skin endothelial cell line (HMEC-1). Cladribine derivatives are less cytotoxic against normal cells, and selectively inhibit the growth of tumor cells. The present results were consistent with those of previous studies^23^. Moreover, the results of spectrophotometric studies in promyelocytic and lymphoblastic leukemia cells performed using the MTT (3–4,5-dimethyltriazo-2-yl-2,5-diphenyltetrazolium bromide) assay were consistent with our results^24^.

The ataxia telangiectasia-mutated *(ATM)* and ATR kinases are the main regulators of the DNA damage response activated by DNA double-strand breaks, and phosphorylate several key proteins that activate the DNA damage checkpoint, DNA repair, and apoptosis or lead to cell cycle arrest^10^. CLA is selectively cytotoxic against acute lymphoblastic leukemia (CCRF-CEM cell line) and HL-60 cells, which have a high level of dCK and low levels of 5′-nucleotidase activity. The effect of this drug is closely related to that of dCK^25–27^. We therefore evaluated the role of ATR kinase in the activation of dCK. Cladribine derivatives activated dCK in acute monocytic, promyelocytic, and lymphoblastic leukemia cells. The highest dCK activity in acute monocytic leukemia cells was observed after incubation with CLA-FMOR and CLA-FPIR derivatives, whereas in acute promyelocytic and lymphoblastic leukemia cells, the highest activity was observed after incubation with a CLA-FMOR derivative. The ATR kinase inhibitor VE-821 decreased dCK activity to control levels. This suggested that in response to genotoxic factors, the ATR kinase inhibitor is active in the absence of Chk-1 phosphorylation. It reduced the level of Ser-74 phosphorylation or the dCK activation site. Our results demonstrated that ATR kinase inhibitor significantly reduced the cytotoxicity of CLA and all tested derivatives. The inhibition of this kinase resulted in the lack of activation of dCK kinase responsible for the phosphorylation of cladribine. This suggests the pro-survival function of this kinase. To assess more directly the role of ATR in the control of dCK activity ATR siRNA should be added before induction of DNA damage by cladribine derivatives. In these conditions, activation of dCK by new derivatives of CLA will be probably suppressed, which would indicate the role of ATR in this process. VE-821 also decreased dCK activity in chronic lymphocytic leukemia cells (EHEB), HL-60 cells, breast cancer cells (MCF-7), and pancreatic cancer cells (PANC-1), indicating that the regulation of dCK activity by ATR was generalized to various cell types^12^. The dCK exists in phosphorylated form under basic conditions because it is constitutively active in cells responsible for the phosphorylation of Ser-74. ATR regulates dCK activity not only in cells with damaged DNA, but also in normal cells and in hematopoietic or epithelial cancer cells^28^. CLA significantly increased the level of ATR and ATM mRNA, which played an important role in dCK kinase phosphorylation^10^.

In the present study, the results obtained using the alkaline version of the comet test indicated that both cladribine and its derivatives caused DNA damage in leukemic cells. Cladribine induced apoptosis and DNA fragmentation in acute lymphoblastic leukemia (CCRF-CEM cell line) and in Burkett’s lymphoma (RAJI cell line)^10^. DNA damaging drugs, such as CLA, induce the formation of DNA-protein complexes, generate single breaks in the DNA strand, and double strand breaks of DNA, as confirmed by qPCR^24^. We confirmed that CLA-FMOR and CLA-FPIP induced the formation of DNA-protein cross-links in THP-1 cells. However, in the case of acute promyelocytic and lymphoblastic leukemia cells, a significant increase in DNA fragmentation was observed after incubation with CLA-FMOR. DNA damage induced by the tested derivatives was significantly higher than that generated by the parent compound. CLA-FPIR and CLA-FMOR derivatives caused greater DNA damage than cladribine, inducing single- and double-stranded DNA breaks and cross-linking in acute monocytic leukemia cells. Similar changes were observed after treatment of acute promyelocytic leukemia cells with a lymphoblastic derivative of CLA-FMOR. ATR kinase inhibitors decrease the levels of DNA damage induced by CLA and derivatives regardless of incubation time^26^. Previous studies showed that cladribine induces ATR-dependent phosphorylation of H2AX, a biomarker for DNA double-strand breaks, and the p53 suppressor protein^12,29^. Another study confirmed that 2-CdA upregulated MRE11A, RAD50, NBN, and decreased CDC25C level significantly^10^. In our study, CLA derivatives increased H2AX level in tested cancer cell lines in a manner dependent on the drug sensitivity of the cells. We show here that γH2AX accumulation, whether induced by new cladribine derivatives was significantly reduced by the ATR inhibitor VE-821.

Modern drugs are designed to induce apoptosis specifically in abnormal cells, leaving healthy cells intact. Purine analogues, including CLA, have several mechanisms of action leading to the induction of apoptosis. Apoptosis, in contrast to necrosis, is not harmful to the host, and does not induce an inflammatory reaction; therefore, it is the preferred pathway of cell death induced by anticancer drugs^30^. Clarification of the mechanisms by which cytotoxic drugs inhibit the proliferation of cancer cells and induce apoptosis is important to optimize therapeutic efficacy. In acute promyelocytic leukemia and lymphoblastic leukemia cells, changes in intracellular calcium levels occurred after treatment with all the tested derivatives^31^. Studies suggest that lymphocytes express L-type Ca^2+^ channels that are activated in a non-voltage-gated manner^32^. The obtained data revealed that compounds 1 and 2 are potent to activate caspase 3, the enzyme which is involved in proteolysis of numerous proteins (e.g. PARP-1) and to activate a key endonuclease. The inhibitor of ATR kinase significantly reduced the level of calcium ions in the cells. This was most likely due to decreased apoptosis in these cells, because the ATR kinase inhibitor downregulated the pro-apoptotic proteins BAX, caspase-3, and PUMA. This suggested a pro-apoptotic function of ATR, which was confirmed by the effect of ATR inhibition on decreasing the cytotoxicity of purine analogues^12^.

Cladribine activates the intrinsic pathway of apoptosis by inducing caspases such as caspase −3, 7, −8, or −9. A decrease in the mitochondrial membrane potential leads to changes in the intracellular levels of calcium^9,33^. The present study clearly showed that the new derivatives significantly increased the levels of calcium ions to a greater extent than CLA. In THP-1 cells, the most notable changes were observed after 2 h of incubation with CLA-FMOR and CLA-FPIR. Cells, after 24 h incubation with compounds, were stained with the Fluo-4-AM probe and visualized under an inverted fluorescence microscope. Cell morphology indicated apoptotic changes like blebbing. However little blebs can be membranous only. The pictures taken do not allow to determine changes at the DNA level. Literature data indicate that besides Ca ^2+^, other pro-apoptotic condition must be met for apoptosis to occur. Apoptosis is associated with DNA cleavage, cell shrinkage, cytoplasmic blebbing and chromatin condensation. Activation of caspase 3 confirmed the nature of the changes induced by the tested compounds.

Another mechanism underlying the effect of cladribine is the induction of cell cycle arrest and condensation of chromosomes. Cladribine leads to the accumulation of cells in the G2/M phase at the expense of the G1 phase^9^. In HL-60 cells, the CLA-FMOR derivative increased the number of cells in the sub-G1 phase. However, in MOLT-4 cells, the tested compounds increased the number of cells in the sub-G1 phase regardless of incubation time and reduced the number of cells in the G1 phase. The CLA-FMOR derivative is responsible for reducing the number of cells in the G1 phase and stops the cell cycle in the S phase. There is a correlation between cell cycle disorders and the sensitivity of THP-1 cells to CLA-FMOR. Incubation with the CLA-FPIR and CLA-FPIP derivatives for 48 h decreased the number of cells in the G1 phase and caused cell cycle arrest in the S-phase. These findings suggested that the new CLA derivatives could be useful for controlling cancer cell growth, as several cancer cells have defects in cell cycle checkpoints. Loss of G1 checkpoint control is common in cancer^34^. Similar results were obtained in other studies^9^.

**Figure 9:**
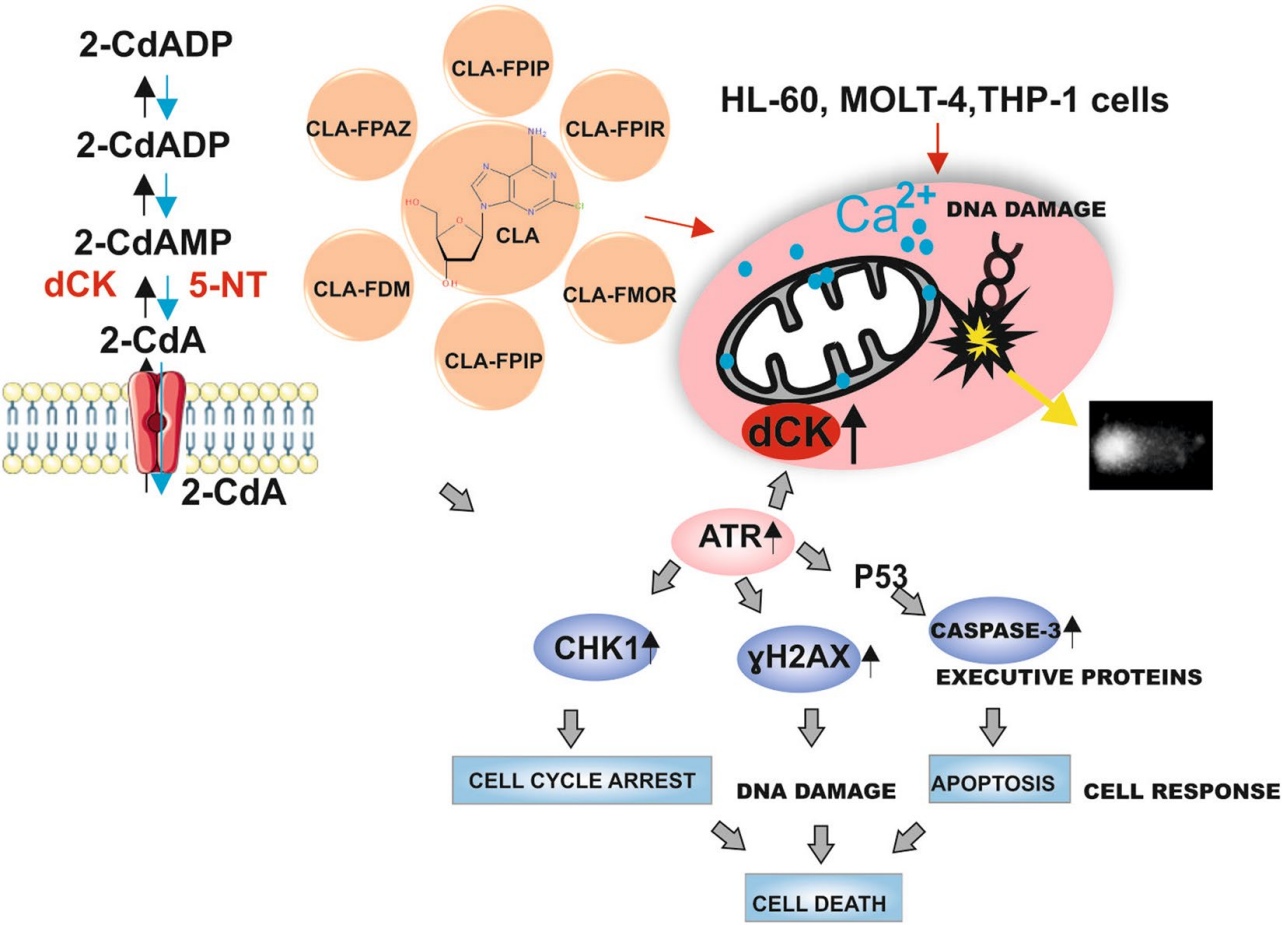
Proposed model of the molecular and cellular responses to the new CLA derivatives. Action of cladribine in leukemia cells: transport of 2-CdA to the cell and the mechanisms underlying the cellular response to genotoxic stress induced by new CLA analogs. DNA strand breaks lead to the activation of the DNA damage response pathway. DNA damage, ssDNA and dsDNA, are recognized by ATR. The consequence of this process is the formation of large clusters of proteins within the DNA damage necessary to activate effector proteins. P53 lead to cell arrest in the cycle and consequently to damage repair or apoptosis.

Taken together, the present results showed that the new cladribine derivatives have high activity against acute monocytic, promyelocytic, and lymphoblastic leukemia cells. They were cytotoxic against leukemic cells and showed low toxicity towards normal cells, which was a highly beneficial result from the point of view of chemotherapy. The tested CLA derivatives significantly increased level of knoc-3/7, histone H2AX and DNA fragmentation including DNA-protein cross-links, caused changes in the intracellular levels of calcium ions, and interfered with the cell cycle. We indirectly confirmed that dCK was a substrate of ATR and demonstrated that ATR played an important role in the regulation of dCK kinase activity in response to DNA damage. Activation of dCK by DNA damage might be mediated by ATR in response to genotoxic agents such as the new CLA derivatives. Nevertheless, further work is needed to determine the respective roles of ATR in the cellular response to new purine analogs. It is desirable to investigate in the future the effect of new derivatives on the mouse model. This will allow not only to prove the efficacy of derivatives but to assess the side effects of the compounds action. Moreover, translation to mouse models efficacy of CLA derivatives compared with standard CLA would be respected. These compounds containing a formamidine group at position 6 may be of clinical significance in the future (Fig. 9).

## Materials and Methods

### Reagents

The deoxycytidine kinase kit was obtained from EIAab (Wuhan, China, Cat no: E8892h). Human H2A.X (phospho S139) In-Cell ELISA Kit was purchase in abcam (Cat no: ab131382). CellEvent™ Caspase-3/7 Green Detection Reagent was from Thermo Fisher Scientifc (Cat no: C10423). The Fluo-4 NW Calcium Assay Kit was obtained from Thermo Fischer Scientific (Waltham, MA, USA, Cat no: F36206). Culture medium (RPMI 1640) and fetal bovine serum (FBS) were obtained from Cambrex (Basel, Switzerland); trypsin-EDTA, penicillin, streptomycin, and VE-821 (Cat no: SML1415) were acquired from Sigma-Aldrich (St. Louis, MO, USA). Other chemicals and solvents were of high analytical grade and were obtained from Sigma-Aldrich or Avantor Performance Materials Poland S.A. (Gliwice, Poland).

### Adenosine derivatives

**Figure 10:**
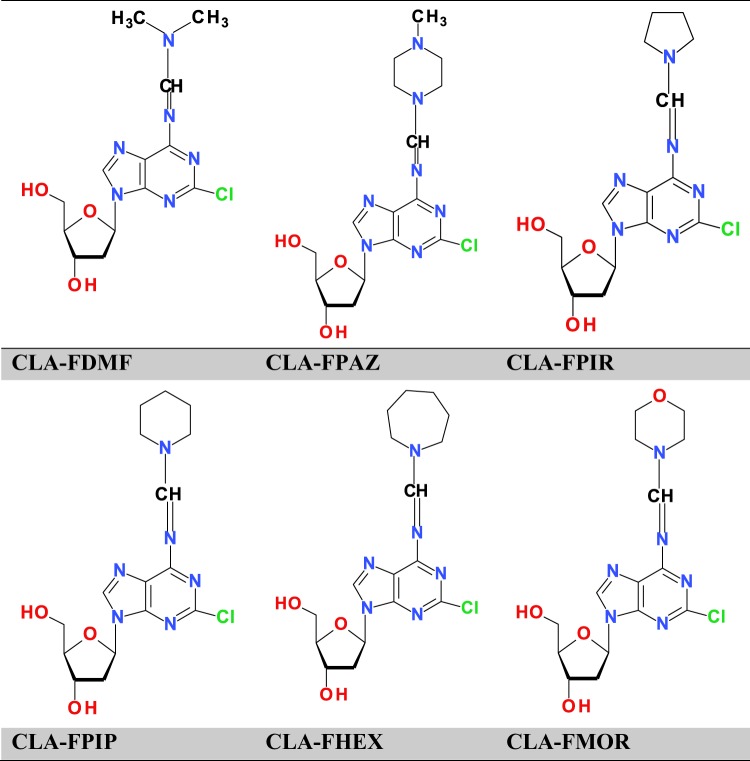
Structure of the tested compounds.

The present study investigated the cytotoxicity and proapoptotic potential of cladribine and six of its derivatives containing a formamidine group at position 6, such as CLA-FDM [6-deamino-6-(N′,N′-dimethyl formamidine)] cladribine, CLA-FPAZ [6-deamino-6 N,N-3′methylase-1′,5′ pentamethylene formamidine)] cladribine, CLA-FPIR [6-deamino-6- (N′, N′-l′, 4′-tetramethyleneformamidino)] cladribine, CLA-FPIP [6-deamino-6- (N′, N′-1′, 5′-pentamethylene formamidine)] cladribine, CLA-FHEX [6-deamino-6- (N′, N′-1′, 5′-pentamethyleneformamidino)] cladribine, and CLA-FMOR [6-deamino-6- (N′, N′-3′-oxa-1′, 5′-pentamethyleneformamidine)] cladribine, synthesized in the Department of Modified Antibiotics at the Institute of Biotechnology and Antibiotics in Warsaw (Fig. 10). The construction of compounds was confirmed by mass spectrometry (MS). MS spectra were acquired on an Applied Biosystems 4800 MALDI TOF/TOF mass spectrometer in MS Reflector Positive Ion Mode, where the matrix was α-cyano-4-hydroxycinnamic acid (CHCA). Samples were prepared using the Dried Droplet Crystallization method. The dialkyl acetals of N, N-disubstituted amides are effective substrates for the synthesis of various trisubstituted amidines. Amidine analogs of cladribine were therefore synthesized by reaction of cladribine with dialkyl acetals. Acetals of disubstituted amides are prepared by converting the appropriate formyl derivative of secondary amines into complexes with dimethyl sulfate, and then treating the resulting complex with sodium methoxide.

### Cell culture

Human HL-60 cells (*Human promyelocytic leukemia cells) were obtained from* the Institute of Biotechnology and Antibiotics (Warsaw, Poland). MOLT-4 cells (*human acute lymphoblastic leukemia cell line*), THP-1 cells (*human leukemia monocytic cells), and HMEC-1 cells (homo sapiens dermal endothelium*) were from the American Type Culture Collection (ATCC; Rockville, MD, USA).

The cells (HL-60, THP-1, MOLT-4) were cultured in RPMI 1640 or MCDB 131 supplemented with L-glutamine 10 mM (HMEC-1). All culture media contained 10% fetal bovine serum, penicillin (10 U/mL), and streptomycin (50 µg/mL), and were regularly checked for mycoplasma contamination. The cells were cultured under an atmosphere of 5% CO_2_ and 95% air at 37 °C. Cladribine as well as its derivatives were synthesized at the Institute of Biotechnology and Antibiotics. Test compounds were dissolved in DMSO. They were stored frozen at −20 °C and divided into small portions. Concentrated drug solutions were thawed immediately before use, diluted in PBS, and added to the cell culture medium at the final concentration.

### Cell cytotoxicity assay

The cytotoxic activity test with XTT (sodium 3′-[1-(phenylaminocarbonyl)- 3,4-tetrazolium]-bis (4-methoxy-6-nitro) benzene sulfonic acid hydrate) is a colorimetric method for determining the parameters associated with cell viability. The cytotoxicity in HL-60, THP-1, MOLT-4, and HMEC-1 cells was measured by the XTT assay. XTT is reduced to formazan by the mitochondrial respiratory chain oxidoreductase, which is active only in living cells. The formed crystals of formazan are dissolved directly in the medium. The amount of product is proportional to the number of live cells.

The IC50 parameter was determined by dividing the mean absorbance values of the drug-treated samples by those of control cells and defined as the drug concentration causing a 50% reduction of cell viability relative to the control. It was calculated using the “Ed50v10(1)” computer application. Cells were plated in 96-well plates at a density of 15 × 10^3^ cells in 100 μL culture medium. Drugs were then added at a concentration range of 10 nM–10,000 nM to obtain a final volume of the mixture of 150 μL. Some cells were preincubated with an ATR kinase inhibitor (VE-821) for 1 h at 37 °C before adding the tested compounds. In this case drugs were added at a concentration range of 10 nM–300 nM. For the XTT assay, after 48 h of incubation, the plates were centrifuged at 1,500 rpm for 10 min at 4 °C, the culture medium was removed, and the cells were suspended in 100 mm^3^ of HBSS (Hank’s Buffered Salt Solution) containing 140 mM NaCl, 5 mM KCl, 0.8 mM MgCl_2_, 1.8 mM CaCl_2_, 1 mM Na_2_HPO_4_, 10 mM HEPES (N- (2-hydroxyethyl) -piperazine-N′- (2-ethanesulfonic acid), and 1% glucose at pH 7.4. Then, 50 μL of XTT solution (in PBS) was added to a final concentration of 1 mg/cm^2^. In addition, PMS (phenazine methosulphate) - methyl dibenzo-N-methylpyrazine sulphate (0.383 mg/cm^3^) was used, which was combined with XTT solution at a ratio of 1:50. After 4 h of incubation with XTT, absorbance was measured at a wavelength λ = 450 nm and reference wavelength λ = 690 nm with a microplate reader (BioTek, Winooski, VT, USA).

### Deoxycytidine kinase

Deoxycytidine kinase activity was measured in accordance with the manufacturer’s protocol. The cells, at final concentration were plated at 1 × 10^6^ cells per 24-well plates and cultured with the tested compounds for 24 h. Some cells were preincubated with an ATR kinase inhibitor (VE-821) for 1 h at 37 °C before adding the tested compounds. After drug treatment, the cells were washed with PBS and resuspended in an ice-cold cytosol extraction buffer containing 1 mM phenylmethylsulfonyl fluoride (PMSF) and a protease inhibitor cocktail. The cell lysate was centrifuged at 10,000 × *g* for 30 min at 4 °C. The protein concentration was determined using the Bradford method. The supernatant (cytosolic fraction) was collected and stored at −80 °C. The clarified cytoplasm extracts, dCK standards, and blank control were added to the appropriate microtiter plate wells with a biotin-conjugated polyclonal antibody prepared specifically for dCK. Avidin conjugated to horseradish peroxidase (HRP) was added to each microplate well and incubated. Then, a TMB substrate solution was added to the plate. Only those wells containing dCK, biotin-conjugated antibody, and enzyme-conjugated Avidin exhibited a change in color. The enzyme-substrate reaction was terminated by addition of a sulfuric acid solution. The color change was measured spectrophotometrically at a wavelength of 450 nm using a microplate reader (BioTek).

### Intracellular calcium assay

Intracellular calcium levels were determined using the fluorescent probe Fluo-4 NW. The cells were plated in 96-well black plates (15 × 10^3^ cells/well) and treated with cladribine and the new derivatives. Incubation with drugs was performed for different times (2–48 h). The medium was then removed, and the cells were washed with phosphate buffered saline (PBS)^35,36^. Finally, a dye loading solution (Fluo-4 NW dye, probenecid, assay buffer—1xHBSS, 20 mM HEPES; 100 μL per well) was processed according to the Fluo-4 NW Calcium Assay Kit protocol (Molecular Probes) and incubated for 30 min in the dark at 37 °C, and then for another 30 min at the room temperature. The measurements were calculated on a fluorescence plate reader for excitation at 494 nm and emission at 516 nm. To avoid errors in the measurement of fluorescence intensity, caused by feasible cell detachment and resulting decrease in the cell number in the samples due to the drug treatment, DNA content was estimated as well.^37^ After the measurement of intracellular calcium fluorescence, the probes were centrifuged at 300xg, 10 min. The cell monolayers were washed three times with PBS and the plate was frozen at −70 °C. Immediately before measurement the microplate with cells was thawed at room temperature, then 100 μl of deionized water was added to the appropriate wells and the microplate with cells was frozen again at −70 °C. After subsequent thawing, RNA was digested with RNAse^37,38^. Then 100 μl of 5 μM of propidium iodide (PI) was added, the plate was immediately shaken, incubated for 15 min at room temperature in the dark and the fluorescence was read at 350/620 nm with a Fluoroskan Ascent FL microplate reader (Labsystems, Sweden).

### Caspase 3/7 assay

The activities of caspases-3 and −7 were estimated with CellEvent™ Caspse-3/7 Green Detection Reagent (Thermo Fisher Scientific) according to the manufacturer’s protocol. The cells were seeded on 96-well plates (15 × 103/ well) and 24 h later, incubated with the appropriate drugs for 24 h or 48 h. The cells were fixed by adding a final concentration of 4% paraformaldehyde solution (10 min, room temperature). Cells were labeled with CellEvent™Caspase-3/7 Green Detection Reagent (5 µM), followed by Hoechst 33342 (5 µg/ml) for 15 minutes in complete medium3. Caspase-3/7 Green Detection Reagent was diluted into PBS with 5% FBS to avoid fluorescence background. After activation of caspase-3/7 in apoptotic cells, the DEVD peptide was cleaved, enabling the dye to bind DNA, which produced a bright, fluorogenic response with absorption/emission maxima of 502/530 nm according with manufacture protocol. Fluorescence intensity was measured using a Fluoroskan Ascent FL plate reader (Labsystem, Sweden). Cysteine protease activity was expressed as a ratio of fluorescence of the drug-treated samples to the corresponding untreated controls (taken as 100%). In some experimental variants, cells were preincubated for 1 h with VE-821 (10 µM) or Z-FA-FMK (caspas-3 inhibitor, 0.5 µM) to confirm that the fluorescence observed in both control and drug-treated cells was due to the presence of caspase-3/7 in samples. The caspase 3/7 signal was normalized to the Hoechst 33342 staining intensity to account for differences in cell seeding density (description in: Intracellular calcium assay section).

### H2AX assay

The level H2AX protein phosphorylated Ser139 was measured in tested leukemia cells by using of In-Cell ELISA, ICE assay kit. The cells were seeded on 96-well plates (100 × 10^3^/well) and 48 h later, incubated with the appropriate compounds. In some experimental variants, cells were preincubated for 1 h with VE-821 (10 µM). The cells were fixed by adding a final concentration of 4% paraformaldehyde solution (10 min, room temperature). After 2 h of Block Solution incubation, cells were treated with a highly specific antibody. A rabbit monoclonal antibody specific to H2AX (phospho S139) was used (at 1:100, 2 h incubation). Next Secondary Antibody Solution (at 1:1000) were added to the cells. The measurements were obtained on a fluorescence plate reader at an excitation wavelength of 680 nm and emission at 694 nm. The cells were PBS washed and 100 µL of Janus Green Stain was added to each well of the plate (5 min., room temperature). The data were collected at excitation wavelength of 750 nm and emission at 800 nm channel (Fluoroskan Cary Eclipse). Antibody signal intensity was normalized to the total amount using Janus Green cell-stain.

### Cell cycle analysis

Cellular DNA content was quantified by flow cytometry. Cells were treated with drugs for 2, 24, and 48 h. For experiments with inhibitors, cells were subjected to 1 h preincubation with VE-821. CLA, CLA-FMOR, CLA-FPIP, or CLA-FPIR was added, and incubation continued for the indicated times under the same conditions. After incubation was completed, cells were collected, washed twice with PBS, and fixed in 70% ethanol. After ethanol fixation (at least 24 h at 4 °C), cells were washed in PBS and centrifuged at 7,000 × *g* for 10 min at 4 °C. Pelleted cells were stained by adding 300 µL PBS containing PI and RNase at final concentrations of 75 µM and 20 µg/mL, respectively. This was followed by 1 h incubation in total darkness at 37 °C. Stained cells were analyzed using a flow cytometer (Becton Dickinson, San Jose, CA, USA). The cell populations at specific phases of the cell cycle were quantified from a standard count of 10,000 cells using FlowJo cytology software (Ashland, OR, USA)^39,40^.

### Comet assay

Single cell gel electrophoresis (comet assay) was used to detect alkali labile sites, single and double DNA strand breaks, and cross-links induced by genotoxic agents. After electrophoresis and gel staining with 4,6-diamidino-2-phenylindole (DAPI), the released DNA was visible under a fluorescence microscope as a characteristic comet tail. The cells were plated on 24-well plates (20,000 cells/mL) and treated with CLA, CLA-FMOR, CLA-FPIP, or CLA-FPIR at the indicated concentrations for 2, 24, and 48 h at 37 °C. Some cells were preincubated with an ATR kinase inhibitor (VE-821) for 1 h at 37 °C before adding the tested compounds. Next, cells were collected into Eppendorf tubes and rinsed with PBS. Then, cells were suspended in 0.75% low melting point (LMP) agarose dissolved in PBS (pH 7.4) and placed on microscope slides precoated with 0.5% normal melting point (NMP) agarose. Subsequently, the slides were treated with a cooled lysis buffer (2.5 M NaCl, 100 mM EDTA, 10 mM Tris, 1% Triton X-100, pH 9.0) for 1–24 h at 4 °C. Then, slides were placed in developing buffer (300 mM NaOH, 1 mM EDTA) for 20 min. Electrophoresis was performed in TAE buffer (30 mM NaOH, 1 mM EDTA) at 29 V and 30 mA for 20 min. The samples were stained with DAPI (1 g/ml). The slides were stored in a wet chamber at 4 °C and analyzed under a fluorescence microscope. Fifty randomly selected cells from each slide were measured using image analysis (Nikon, Japan) attached to a COHU 4910 video camera, which was equipped with a UV-1 filter block consisting of an excitation filter (359 nm) and a barrier filter (461 nm) connected to the image analysis system Lucia-Comet v. 4.51 (Czech Republic)^41^. In the samples preincubated with VE-821, the level of DNA damage associated with the ATR kinase was determined.

### Detection of DNA-protein cross-links

Because cross-links can be undetectable in the alkaline comet assay, a variant of this method including post-treatment with proteinase K (Cat no: P2308) was used. Proteinase K digests peptide bonds, releasing the cross-linked DNA into the DNA-protein complexes without altering the structure of the DNA-DNA complexes to distinguish them. After lysis, the slides were washed three times in TE buffer (10 mM Tris, pH 10, 1 mM EDTA). The cells were treated with aliquots of proteinase K (60 µL per slide; 1 mg/mL TE buffer) for 1 h at 37 °C in a wet chamber^40^. Further steps of the comet assay were performed as described above.

### Statistical analysis

The data are presented at least as the mean ± SD of three independent experiments. ANOVA was performed with the Tukey post hoc test for multiple comparisons (StatSoft, Tulsa, OK, USA)^39^. The significance level was set at a P value of 0.05.
